# Translation and cultural adaptation of the Pregnancy Physical Activity Questionnaire into Danish using the dual-panel approach: comparison with outcomes from an alternative translation approach

**DOI:** 10.1186/s13104-021-05640-6

**Published:** 2021-06-03

**Authors:** Jannie Tygesen Schmidt, Josephine Nielsen, Allan Riis, Birgit Tine Larsen

**Affiliations:** 1 Department of Physiotherapy, University College of Northern Denmark, Selma Lagerloefs Vej 2, 9220 Aalborg, Denmark; 2 Center for General Practice at Aalborg University, 9220 Aalborg, Denmark

**Keywords:** Pregnancy, Exercise, Sedentary behaviour, Patient Reported Outcome Measure, Women’s Health Services

## Abstract

**Objective:**

Physical activity reduces the risk of pregnancy-related complications. However, pregnant women often reduce their physical activity levels and do not follow the WHO’s physical activity recommendations during pregnancy. To support pregnant women in monitoring physical activity, the self-administered Pregnancy Physical Activity Questionnaire was developed in the US. We translated and cross-cultural adapted the questionnaire using the dual approach method. Meanwhile, and without knowing this, another Danish group simultaneously translated the questionnaire using the method described by Beaton et al. The aim is to present our data and discuss the unplanned purpose of comparing the results from using two different translation methods.

**Results:**

We translated and cross-culturally adapted the Pregnancy Physical Activity Questionnaire to Danish with the following findings. Two additional items for cycling were included. Three items about spending time on a computer, reading, writing or talking on the phone were not feasible in terms of differentiating between them and these were merged into one item. The item ‘Taking care of an older adult’ was found to be irrelevant in a Danish setting and was removed. Adaptions were similar comparing the two methods. Consequently, using the dual-panel and the methods suggested by Beaton et al. yield similar results when translating and cultural adapting the PPAQ.

**Supplementary Information:**

The online version contains supplementary material available at 10.1186/s13104-021-05640-6.

## Introduction

The objective of this study was to report on the translation and cultural adaptations of a questionnaire used for research purposes. We have translated and culturally adapted the original US version of the Physical Pregnancy Activity Questionnaire (PPAQ) to a Danish version among a population in the Region of Northern Denmark. Translation and cultural adaptation of the PPAQ following the dual-panel approach [1] is referred to as PPAQ-DK2 in this manuscript.

However, meanwhile, a research group at the University of Copenhagen and Nordsjaellands Hospital in Denmark also carried out a similar project using translation methods described by Beaton et al. [2] translating the questionnaire to Danish (PPAQ-DK) [3]. The two research groups were not aware of each other’s work. The PPAQ-DK was published 8th December 2020. In this research note, we report on the unpublished findings of the translation and cultural adaptation of PPAQ-DK2 study from the University College in the Region of Northern Denmark and discuss the differences and commonalities in the conclusions despite using different methods of translation and cultural adaptations.

## Main text

Previous studies have found that women who are active during pregnancy reduce the risk of lifestyle-related pregnancy complications such as gestational diabetes, preeclampsia and risk of preterm birth [4–6]. However, lifestyle-related pregnancy complications frequently occur because of physical inactivity and obesity [7]. The self-administered Pregnancy Physical Activity Questionnaire was developed in the US (PPAQ) to support pregnant women in monitoring their level of activity. The PPAQ is found to be reliable and is currently considered among the best available tools to assess physical activity (PA) during pregnancy [8]. The PPAQ has been widely translated into several languages, e.g., Chinese, Spanish, Turkish, Brazilian, and Vietnamese [9–13]. Hence, this study aimed at translating and culturally adapting the PPAQ into Danish. At that time, the PPAQ had not yet been translated into Danish.

The questionnaire measures average physical activity. The PPAQ includes 36 items in total, i.e., three items on the date of completion of the questionnaire and dates relevant to the pregnancy’s progress followed by 33 items on time spent in various activities [14]. Hence, the PPAQ distinguishes between day-to-day activities such as cooking and household chores, recreational sport (e.g., jogging and dancing), and childcare (nursing and playing). The 33 items concerning activities are divided into 13 household/caregiving activities, 5 occupational activities, 9 sports/exercise activities, 3 questions regarding physical inactivity, including the possibility of adding activities not already included in the questionnaire if the respondent finds it relevant, and 3 questions regarding transportation activities [14]. For each item, participants select a category of the amount of time spent carrying out each specific activity ranging from 0 to 6 or more hours/day (h/day) or from 0 to 3 or more hours/week (h/wk.) [14]. Subsequently, the amount of time spent carrying out each activity is converted into the Metabolic Equivalent of Task (MET), which describes the physical activity levels measured in metabolic equivalents i.e., relative to the energy expenditure during rest [15]. According to the calculation guideline by Chasan-Taber et al. [16], each item refers to a given MET value.

The translation of the PPAQ into PPAQ-DK2 was made using the dual-panel approach, which divides the translation process into three steps [1]. The first panel of seven translators reached a consensus on all items and drew attention to 4 items specifically for the second panel to decide upon. The second layman panel of nine non-pregnant women agreed that three items should be merged into one item. Two items regarding commuting by bike should be added and the panel suggested rephrasing one item regarding jogging. In step three (the target group panel), 21 pregnant women filled out the questionnaire.

**Fig. 1:**
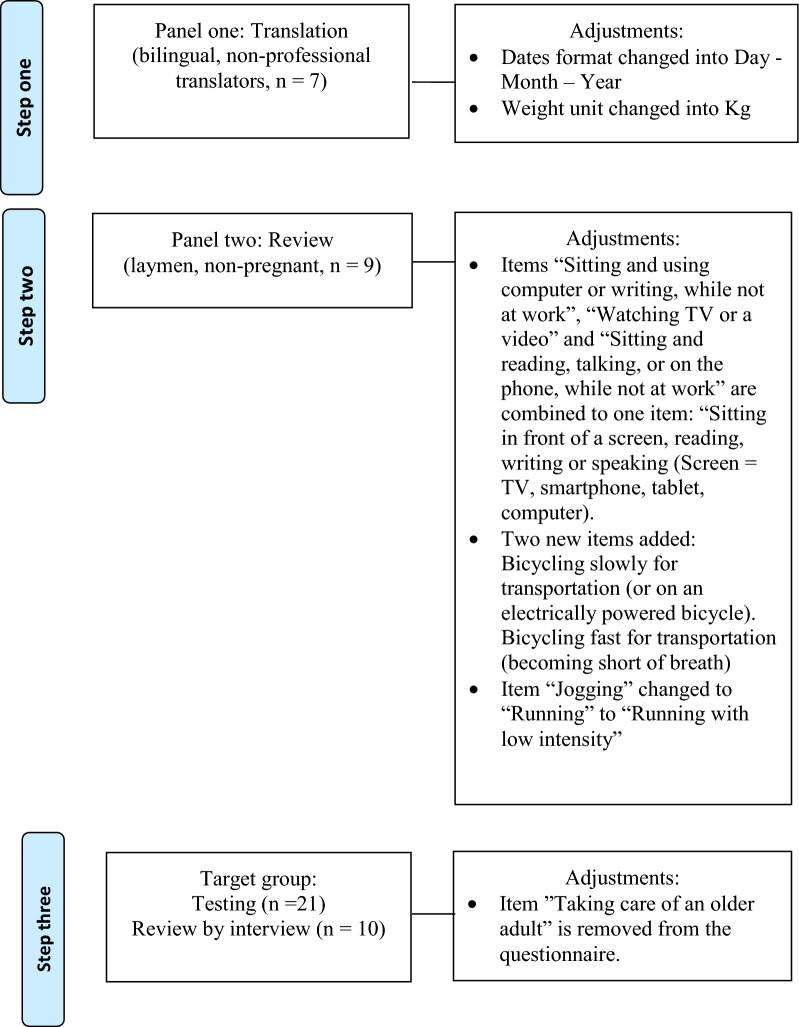
Adjustments to the PPAQ items during the dual-panel translation process in PPAQ-DK2. This figure illustrates the flow of adaptations of the Physical Pregnancy Activity Questionnaire during the three-step dual-panel translation process

21 filled out the Danish version of the questionnaire with the researchers present. Following this, a subsample (n = 10) were randomly selected and interviewed through semi-structured single-person interviews (Table 1) and all informants found the questionnaire to be comprehensible, relevant to them and easy to fill in. Finally, to assess face validity, they were asked if they found the questionnaire relevant to them in their current situation. The need for two additional items about cycling was identified. The questionnaire was subsequently revised accordingly by including two items on slow and fast cycling, respectively, and “let tempo” (low intensity) was added to the translation of the item “Jogging” (Fig. 1). Compared to US, in Denmark cycling is custom and cycling is frequently used as means of transportation but also for exercise purposes. Furthermore, a differentiation between the three items “Sitting and using a computer or writing while not at work”, “Watching TV or a video” and “Sitting and reading, talking or on the phone while not at work” was not found relevant. These activities were often conducted using the same devices. Furthermore, reading activities and watching videos was not always considered two divergent activities. Consequently, the three items were combined into one.

**Table 1:** Characteristics of the study population

	(n = 21, all)	(n = 10, interviewees)
Age (years), mean (SD)	29.1 (3.7)	29.4 (4.4)
College degree, number (%)	15 (71.4)	7 (70.0)
Week of pregnancy, median (IQR)	34 [29–36]	35 [22–38]

N = 21, All filled out the Danish version of the questionnaire, among these a subsample participated with interviews

College degree: graduate of an educational institution (school, college, university)

Week of pregnancy: pregnancy is counted as 40 weeks, starting from the first day of the mother’s last menstrual period

*SD* standard deviation, *IQR* interquartile range

Following step three, a final adjustment was made to the questionnaire and the item ‘Taking care of an older adult’ was removed, as all 21 responders replied “none”, i.e., no time was spent on the item and several informants commented on the lack of relevance of the item to their life situation (Fig. 1). This finding is in line with the stated goals for elderly care in Denmark. Where, home nursing services are provided by the municipalities. All citizens are entitled to home nursing free of charge when prescribed by a medical doctor and based on an assessment of an individual need [17]. The material and methods are further described and the final version of PPAQ-DK2 is included in Additional file 1.

The research group at the University of Copenhagen and Nordsjaellands Hospital in Denmark also translated and culturally adapted the PPAQ applying the five stages as recommended by Beaton et al. [2]. The two research groups being unaware of each other work with the PPAQ.

In contrast to the method suggested by Beaton et al., the dual-panel approach involves gathering two panels and arranging for two meetings, which can be a practical challenge. Also, it requires a person with skills in facilitating the discussions by the panels. Although the method suggested by Beaton et al. may be easier to facilitate and is maybe more structured, it requires more steps and lacks the synergy arising from using panels. Beaton et al. include five steps, i.e., in step one, two bilingual translators, whose mother tongue is the target language (in this case Danish), forward translate the questionnaire into the target language and each translator provides a written report. The translators have different profiles or backgrounds. In step two, the two translators meet and synthesise their reports. In step three, working from the synthesised version and unaware of previous versions, a minimum of two translators translate the questionnaire back to the source language. In this process, the translators’ first language is the source language (in this case English). Each translator provides a written report. In step four, all reports are reviewed, and a written report is made. In step five, the pre-final version from step four is pretested by interviewing 30–40 representatives from the target population. This to probe about what they thought was meant by each questionnaire item and the chosen response. Here, both the meaning of the items and responses were explored.

Similar to the results of the dual-panel method in PPAQ-DK2, the item ‘Taking care of an older adult’ was removed, the sedentary items were combined and an item on cycling as transportation was added. Furthermore, one additional item was removed during the five-stage translation process, i.e. ‘Mowing lawn while riding a mower’ and an item on ‘Needlework’ was added.

The application of the two methods for translation and cultural adaptation of the PPAQ resulted in almost similar adjustments to the original US version. Therefore, this research note supports that the dual-panel method and the five-stage method suggested by Beaton et al. were both applicable for translations and cultural adaptations of the PPAQ. We do not know if this is the case for other patient-reported questionnaires.

## Limitations

We translated and culturally adapted the US version of the PPAQ questionnaire into Danish. However, we did not perform additional validation or tests for reliability. Not being aware of two parallel translation processes and comparing the methods afterwards is a limitation when discussing the difference between the two methods for cultural adaptation and translation. Designing a study to compare the two methods could have allowed for more standardised protocols in terms inclusion criteria of respondents and translators. Meanwhile, this ‘natural experiment’ gave a unique chance to compare two frequently used methods for the translation and cultural adaptation of questionnaires.

## Supplementary Information

**Additional file 1.** Complete reporting of the translation and cross-cultural adaption of the Pregnancy Physical Activity Questionnaire into Danish.

## Data Availability

The data used and/or analysed during the current study is available from the corresponding author upon reasonable request. A complete description of the translation of PPAQ-DK2 is included in Additional file 1.

## Abbreviations

PA: Physical activity
PPAQ: The original Physical Pregnancy Activity Questionnaire
PPAQ-DK: The Danish version of the Physical Pregnancy Activity Questionnaire from the capital region of Denmark
PPAQ-DK2: The Danish version of the Physical Pregnancy Activity Questionnaire from University College of the northern region of Denmark (Additional file)
MET: Metabolic Equivalent of Task
