# Supplementary material for: Translation and cultural adaptation of the Pregnancy Physical Activity Questionnaire into Danish using the dual-panel approach: comparison with outcomes from an alternative translation approach
Source: BMC Res Notes. 2021 Jun 3;14:225. doi: 10.1186/s13104-021-05640-6 (PMC8176740; doi:10.1186/s13104-021-05640-6)
Supplement: Supplementary file 1 — Additional file 1. Complete reporting of the translation and cross-cultural adaption of the Pregnancy Physical Activity Questionnaire into Danish. [file 13104_2021_5640_MOESM1_ESM.docx]

Additional file 1. Complete reporting of the translation and cross-cultural adaption of the Pregnancy Physical Activity Questionnaire into Danish

# Introduction

Regular physical activity (PA) can reduce the risk of diabetes, cardiovascular disease, mental disorders and pregnancy-related complications (1) (2,3). Epidemiologic studies have found that active women during pregnancy reduce the risk of lifestyle-related pregnancy complications and their risk of preterm birth (4–6). However, lifestyle-related pregnancy complications such as gestational diabetes and preeclampsia frequently occur because of physical inactivity and obesity (7).

A study in 2015 showed that there is a large percentage of Danish pregnant women in the first trimester who do not meet the National Board of Health's recommendations for PA during pregnancy (8). In addition, PA among pregnant women tends to decrease in duration, with lower frequency and lower intensity relative to pre-pregnancy levels (9). The exact level of physical activity required to reach favourable pregnancy outcomes is unknown (10). However, guidelines for PA throughout pregnancy recommend that healthy pregnant women are active for at least 150 minutes with moderate-intensity exercise each week throughout their pregnancy (10).

To support pregnant women in monitoring their level of activity, the self-administered Pregnancy Physical Activity Questionnaire (PPAQ) was developed in the US. PPAQ was initially used in an intervention trial to measure PA levels among pregnant women and to prevent pregnancy complications (11). However, it is also considered feasible for research purposes and health education during pregnancy (12). The PPAQ is generally found to be reliable and is currently considered among the best available tools to assess PA during pregnancy (13).

PPAQ has been widely translated into several languages e.g. Japanese, Chinese, Polish, Spanish, Turkish, Brazilian, Portuguese, Brazilian, and Vietnamese. Still, it is yet to be translated into Danish. Hence, this study aimed to translate and culturally adapt the PPAQ into Danish.

# Materials and methods

## Ethics

This study was conducted according to the Declaration of Helsinki (14). Approval by the Ethics Committee according to Danish law was not required. Before enrolment, all participants provided written informed consent. The Danish translation of the questionnaire was conducted in agreement with the authors of the original questionnaire.

## Pregnancy Physical Activity Questionnaire

The PPAQ is a self-administered questionnaire to be completed by pregnant women. The questionnaire measures physical activity levels during an average day or week within the specific trimester. The PPAQ includes 36 items in total i.e., 3 items on the date of completion of the questionnaire and dates relevant to the pregnancy's progress, followed by 33 items on time spent on various activities (11). Hence, the PPAQ distinguishes between day-to-day activities such as cooking and household chores, sport for fun (e.g. jogging and dancing), and childcare (nursing and playing). The 33 items concerning activities are divided into 13 household/caregiving activities, 5 occupational activities, 9 sports/exercise activities, including 2 open-ended questions, 3 transportation activities, and 3 questions regarding physical inactivity (11). In addition, activities can be added in the questionnaire if the respondent finds this to be relevant. For each item, participants select a category for the amount of time spent in each specific activity, ranging from 0 to 6 or more hours/day (h/day) or from 0 to 3 or more hours/week (h/wk) (11). Subsequently, the amount of time spent in each activity is converted into a Metabolic Equivalent of Task (MET), which describes the physical activity levels measured in metabolic equivalents i.e., relative to the energy expenditure during rest (15). According to the calculation guideline by Chasan-Taber et al. (16), each item refers to a given MET value. The MET values divide the respective items into sedentary (<1.5 METs), light (1.5–3.0 METs), moderate (3.0–6.0 METs), and vigorous activity (>6.0 METs) (16). Time spent on each item/activity is multiplied by its intensity to arrive at an average weekly energy expenditure (MET-h∙week^-1^) attributable to each activity (16). Finally, total activity and activities of light intensity and above are added together to estimate average MET hours per week for total energy expenditure (11,16).

## Translation process and recruitment

The translation of the PPAQ into Danish was carried out using the dual-panel approach, which divides the translation process into three steps (17).

In step one, a first panel was selected among bilingual nonprofessional translators having a minimum of 7 years of English education and a master's degree requiring experience in academic writing and reading in English. All panel members were informed about the original questionnaire and its intended use before translating the questionnaire into the first Danish version (17). The questionnaire was translated during a group discussion. In the event of any disagreements that were not solved during the group discussions, the debate was noted and a subsequent decision was made by the second panel in step two. Recommendations on the optimal panel size were followed in both step one and two to create favourable conditions for the group discussions (17). In step two, the translated Danish first version of the PPAQ (PPAQ-DK2) was presented to the second panel consisting of laypeople outside of the target group and without health professional degrees. The second panel reviewed and revised the translated version. It was ensured that the participants in the second panel did not know the original questionnaire. This was to ensure that the participants were not affected by what they thought the translated items should mean based on the original wording but preferably on how they interpreted the translated version. A coordinator (JN) and a referent (JTS) were present at both panel discussions to ensure that the conceptual equivalence of the items was maintained throughout the translation process. The coordinator presented the second panel with the alternative wordings that the first panel was not able to agree upon and the referent documented the process.

Finally, in step three, after a review by the second panel, the PPAQ-DK2 was presented to a convenient number of the target population (pregnant women) at the local centre for midwifery. At first, the informants filled out the Danish version of the questionnaire with the researchers (JTS and JN) present. The informants were encouraged to fill out the questionnaire independently, but they could ask the researchers if they had any questions about the it. Following this, a subsample was randomly selected and the pregnant women were interviewed through semi-structured single-person interviews. Sampling was based on the information power to reach data saturation, thereby aiming to discover the population's perspectives (17). The interviewees were asked about general comprehensibility and if they found items difficult to comprehend and/or respond to. Also, they were asked how easy it was for them to fill in the questionnaire. Finally, to assess face validity, they were asked if they found the questionnaire and the questions relevant to them in their current situation.

## Statistical analysis

Descriptive statistics include numbers (%) for categorical variables and mean (SD) or median (IQR) for continuous variables, depending on the distribution of data. Statistics were performed in STATA version 16.0 (StataCorp, College Station, Texas 77845 USA).

# Results

The first panel in step one included seven participants (four female) recruited at the physiotherapy department at the University College of Northern Denmark. All held a health professional degree and an academic master's degree or PhD. The participants were bilingual (Danish and English) ranging from 29 to 63 years. The translation of the PPAQ in step one lasted for one hour and 45 min., and the first panel reached a consensus on all items.

The date format for the first three items in the original PPAQ was modified by converting the order month/day/year into the Danish standard order, which is day/month/year. Likewise, in two items, the metric system was converted from gallons to kilograms (Figure 1). None of these changes were subsequently questioned or commented on in step two and three.

The first panel found it difficult to differentiate between the time spent in the three sedentary items, i.e. items "Sitting and using a computer or writing while not at work", "Watching TV or a video" and "Sitting and reading, talking or on the phone while not at work"(11). Therefore, the first panel requested that the layman panel in step two draw specific attention to these items. The first panel also recommended adding items related to cycling, since this is used for exercise and as a means of transportation by many Danes. Finally, the first panel requested the laymen panel to address if the Danish word" Løb" was an optimal term to describe the item 'Jogging' in the original questionnaire.

The layman panel in step two included nine non-pregnant women between 21 to 35 years of age. They were either skilled workers or held a master's degree and were unaware of the original questionnaire. The panel discussions lasted 55 minutes, during which time the panel agreed to combine the three items "Sitting and using a computer or writing while not at work", "Watching TV or a video" and "Sitting and reading, talking or on the phone while not at work"(11). The panel also agreed to create two additional items for cycling. Finally, the second panel decided that the Danish word "Løb" was the best translation for the item "Jogging" in combination with the adjective "let tempo" (low intensity) to emphasise the desired intensity. The questionnaire was subsequently revised accordingly by including two additional items on slow and fast cycling, respectively, and adding "let tempo" (low intensity) to the translation of the item "Jogging". The changes made during the three steps of the translation process appear in figure 1.

In step three, the target group consisting of 21 pregnant women filled in the questionnaire. Subsequently, 10 women were interviewed to obtain data saturation on the comprehensibility of the updated second version of the PPAQ-DK2 (Table 1).

Participants in step three completed the questionnaire within an average of 10 minutes. All interviewees in the target population found the second version of the questionnaire comprehensible, relevant to their situation, and easy to fill out.

When asked about items "Sitting and using a computer or writing while not at work", "Watching TV or a video" and" Sitting and reading, talking or on the phone while not at work" as it appeared in the first version of the PPAQ-DK2, the interviewees found that a differentiation between the three items was pointless. They preferred to merge the three items into one as appearing in the second Danish version (Figure 1).

All the responders in the target group wrote "none", i.e. no time was spent on the item "Taking care of an older adult" and several informants commented on the lack of relevance of this item to their situation. Consequently, following step three, a final adjustment was made to the questionnaire and the item was removed (Figure 1). The final version of the Northern region of Denmark version (PPAQ-DK2).

**Spørgeskema omhandlende fysisk aktivitet under graviditet**

**Pregnancy Physical Activity Questionnaire Danish edition (PPAQ-DK2)**

**Samtykkeerklæring til deltagelse:**

Jeg ved, at det er frivilligt at deltage i projekt Aktiv og Gravid. Jeg giver med min tilladelse til, at de indsamlede oplysninger må opbevares og benyttes til forskning i 5 år, hvorefter data slettes.

​​☐​ JA  ​☐​ NEJ

**Baggrundsinformation:**

1. Dags dato: : ​☐​​☐​/​☐​​☐​/​☐​​☐​​☐​​☐​ (dd/mm/yyyy)

1. Hvornår var den første dag i din sidste menstruation: ​☐​​☐​/​☐​​☐​/​☐​​☐​​☐​​☐​ (dd/mm/yyyy)

​​☐​ Ved ikke

1. Hvornår har du termin? : ​☐​​☐​/​☐​​☐​/​☐​​☐​​☐​​☐​ (dd/mm/yyyy)

​​☐​ Ved ikke

Instruktioner:

*Udfyld venligst spørgeskemaet med en blå eller sort kuglepen. Sæt kryds i kassen.*

*Det er vigtigt du svarer ærligt omkring dig selv. Der er ingen rigtige eller forkerte svar. Vi ønsker blot at vide noget om de aktiviteter du foretager dig i dit nuværende trimester.****Vi sætter pris på dine svar og din tid.***

*Hvis du ønsker at ændre dit svar, udfyld da kassen fuldstændigt og angiv et nyt kryds. Herunder vises et eksempel:*

*___________________________________________________________________________*

***Eksempel:****Hvis du tager dig af din mor 2 timer hver dag bør din besvarelse se ud som dette:*

*I dit nuværende trimester, når du ikke er på arbejde, hvor meget tid bruger du sædvanligvis på at…:*

*E1. …tage dig af en voksen person*

*​​☐​Ingen*

*​​☐​Mindre end 1/2 time om dagen*

*​​☐​1/2 til1 time om dagen*

*​​☒​1 til 2 timer om dagen*

*​​☐​2 til 3 timer om dagen*

*​​☐​3 eller flere timer om dagen*

I dit nuværende trimester, når du ikke er på arbejde, hvor meget tid bruger du sædvanligvis på at…:

1. … forberede måltider (lave mad, dække bord, vaske op)

​​☐​Ingen

​​☐​Mindre end 1/2 time om dagen

​​☐​1/2 til1 time om dagen

​​☐​1 til 2 timer om dagen

​​☐​2 til 3 timer om dagen

​​☐​3 eller flere timer om dagen

1. … påklæde, bade, made børn(ene) mens du er siddende

​​☐​Ingen

​​☐​Mindre end 1/2 time om dagen

​​☐​1/2 til1 time om dagen

​​☐​1 til 2 timer om dagen

​​☐​2 til 3 timer om dagen

​​☐​3 eller flere timer om dagen

1. … påklæde, bade, made børn(ene) mens du er stående

​​☐​Ingen

​​☐​Mindre end 1/2 time om dagen

​​☐​1/2 til1 time om dagen

​​☐​1 til 2 timer om dagen

​​☐​2 til 3 timer om dagen

​​☐​3 eller flere timer om dagen

1. … lege med børn(ene) mens du sidder eller står

​​☐​Ingen

​​☐​Mindre end 1/2 time om dagen

​​☐​1/2 til1 time om dagen

​​☐​1 til 2 timer om dagen

​​☐​2 til 3 timer om dagen

​​☐​3 eller flere timer om dagen

1. ... lege med børn(ene) mens du går eller løber

​​☐​Ingen

​​☐​Mindre end 1/2 time om dagen

​​☐​1/2 til1 time om dagen

​​☐​1 til 2 timer om dagen

​​☐​2 til 3 timer om dagen

​​☐​3 eller flere timer om dagen

1. … løfte børn

​​☐​Ingen

​​☐​Mindre end 1/2 time om dagen

​​☐​1/2 til1 time om dagen

​​☐​1 til 2 timer om dagen

​​☐​2 til 3 timer om dagen

​​☐​3 eller flere timer om dagen

1. … sidde foran en skærm, læse, skrive og tale

(skærm = TV, smartphone, tablet, computer o.l.)

​​☐​Ingen

​​☐​Mindre end 1/2 time om dagen

​​☐​1/2 til1 time om dagen

​​☐​1 til 2 timer om dagen

​​☐​2 til 3 timer om dagen

​​☐​3 eller flere timer om dagen

1. … lege med kæledyr

​​☐​Ingen

​​☐​Mindre end 1/2 time om dagen

​​☐​1/2 til1 time om dagen

​​☐​1 til 2 timer om dagen

​​☐​2 til 3 timer om dagen

​​☐​3 eller flere timer om dagen

1. … gøre let rent (rede senge, ordne vasketøj, stryge og rydde op)

​​☐​Ingen

​​☐​Mindre end 1/2 time om dagen

​​☐​1/2 til1 time om dagen

​​☐​1 til 2 timer om dagen

​​☐​2 til 3 timer om dagen

​​☐​3 eller flere timer om dagen

1. … handle dagligvarer, tøj mm.

​​☐​Ingen

​​☐​Mindre end 1/2 time om dagen

​​☐​1/2 til1 time om dagen

​​☐​1 til 2 timer om dagen

​​☐​2 til 3 timer om dagen

​​☐​3 eller flere timer om dagen

1. … gøre hovedrent (støvsuge, gulvvask, feje og vaske vinduer)

​​☐​Ingen

​​☐​Mindre end 1/2 time om ugen

​​☐​1/2 til1 time om ugen

​​☐​1 til 2 timer om ugen

​​☐​2 til 3 timer om ugen

​​☐​3 eller flere timer om ugen

1. … slå græs med havetraktor

​​☐​Ingen

​​☐​Mindre end 1/2 time om ugen

​​☐​1/2 til1 time om ugen

​​☐​1 til 2 timer om ugen

​​☐​2 til 3 timer om ugen

​​☐​3 eller flere timer om ugen

1. … slå græs med græsslåmaskine, rive eller øvrigt havearbejde.

​​☐​Ingen

​​☐​Mindre end 1/2 time om ugen

​​☐​1/2 til1 time om ugen

​​☐​1 til 2 timer om ugen

​​☐​2 til 3 timer om ugen

​​☐​3 eller flere timer om ugen

Når du er ude af huset...

I dit nuværende trimester, hvor meget tid bruger du sædvanligvis på at…:

1. … gå langsomt som transport, eksempelvis til bussen, arbejde, besøg. (ikke som motion)

​​☐​Ingen

​​☐​Mindre end 1/2 time om dagen

​​☐​1/2 til1 time om dagen

​​☐​1 til 2 timer om dagen

​​☐​2 til 3 timer om dagen

​​☐​3 eller flere timer om dagen

1. … gå hurtigt som transport, eksempelvis til bussen, arbejde, besøg. (ikke som motion)

☐Ingen

☐Mindre end 1/2 time om dagen

☐1/2 til1 time om dagen

☐1 til 2 timer om dagen

☐2 til 3 timer om dagen

☐3 eller flere timer om dagen

1. … køre i bil eller bus

☐Ingen

☐Mindre end 1/2 time om dagen

☐1/2 til1 time om dagen

☐1 til 2 timer om dagen

☐2 til 3 timer om dagen

☐3 eller flere timer om dagen

1. ... cykle langsomt som transport (eller på eldreven-cykel)

☐Ingen

☐Mindre end 1/2 time om dagen

☐1/2 til1 time om dagen

☐1 til 2 timer om dagen

☐2 til 3 timer om dagen

☐3 eller flere timer om dagen

1. … cykle hurtigt som transport (hvor du bliver forpustet)

☐Ingen

☐Mindre end 1/2 time om dagen

☐1/2 til1 time om dagen

☐1 til 2 timer om dagen

☐2 til 3 timer om dagen

☐3 eller flere timer om dagen

For fornøjelse og som motion...

I dit nuværende trimester, hvor meget tid bruger du sædvanligvis på at:

1. … gå i langsomt tempo

☐Ingen

☐Mindre end 1/2 time om ugen

☐1/2 til1 time om ugen

☐1 til 2 timer om ugen

☐2 til 3 timer om ugen

☐3 eller flere timer om ugen

1. … gå i moderat tempo

☐Ingen

☐Mindre end 1/2 time om ugen

☐1/2 til1 time om ugen

☐1 til 2 timer om ugen

☐2 til 3 timer om ugen

☐3 eller flere timer om ugen

1. … gå i hurtigt tempo (hvor du bliver forpustet)

☐Ingen

☐Mindre end 1/2 time om ugen

☐1/2 til1 time om ugen

☐1 til 2 timer om ugen

☐2 til 3 timer om ugen

☐3 eller flere timer om ugen

1. … løbe i let tempo

☐Ingen

☐Mindre end 1/2 time om ugen

☐1/2 til1 time om ugen

☐1 til 2 timer om ugen

☐2 til 3 timer om ugen

☐3 eller flere timer om ugen

1. … gå på motionshold for gravide

☐Ingen

☐Mindre end 1/2 time om ugen

☐1/2 til1 time om ugen

☐1 til 2 timer om ugen

☐2 til 3 timer om ugen

☐3 eller flere timer om ugen

1. … svømme

☐Ingen

☐Mindre end 1/2 time om ugen

☐1/2 til1 time om ugen

☐1 til 2 timer om ugen

☐2 til 3 timer om ugen

☐3 eller flere timer om ugen

1. … danse

☐Ingen

☐Mindre end 1/2 time om ugen

☐1/2 til1 time om ugen

☐1 til 2 timer om ugen

☐2 til 3 timer om ugen

☐3 eller flere timer om ugen

Laver du andre aktiviteter for fornøjelse eller som motion?

1. Hvilken aktivitet?  ___________________________

☐Ingen

☐Mindre end 1/2 time om ugen

☐1/2 til1 time om ugen

☐1 til 2 timer om ugen

☐2 til 3 timer om ugen

☐3 eller flere timer om ugen

1. Hvilken aktivitet? ___________________________

☐Ingen

☐Mindre end 1/2 time om ugen

☐1/2 til1 time om ugen

☐1 til 2 timer om ugen

☐2 til 3 timer om ugen

☐3 eller flere timer om ugen

*Venligst udfyld den næste sektion hvis du har lønnet arbejde, laver frivilligt arbejde på fuldtid eller er studerende. Hvis du er hjemmegående, på barsel, ikke er i arbejde eller ikke har mulighed for at være i arbejde, skal du ikke udfylde den sidste sektion.*

På arbejde…

I dit nuværende trimester, hvor meget tid bruger du sædvanligvis på at:

1. … sidde på arbejde eller til undervisning (stillesiddende arbejde)

☐Ingen

☐Mindre end 1/2 time om dagen

☐1/2 til1 time om dagen

☐1 til 2 timer om dagen

☐2 til 3 timer om dagen

☐3 eller flere timer om dagen

1. … stå eller gå langsomt mens du løfter noget, som er tungere end 4 kg

☐Ingen

☐Mindre end 1/2 time om dagen

☐1/2 til1 time om dagen

☐1 til 2 timer om dagen

☐2 til 3 timer om dagen

☐3 eller flere timer om dagen

1. … stå eller gå langsomt uden at løfte noget

☐Ingen

☐Mindre end 1/2 time om dagen

☐1/2 til1 time om dagen

☐1 til 2 timer om dagen

☐2 til 3 timer om dagen

☐3 eller flere timer om dagen

1. … gå hurtigt mens du bærer noget som er tungere end 4 kg

☐Ingen

☐Mindre end 1/2 time om dagen

☐1/2 til1 time om dagen

☐1 til 2 timer om dagen

☐2 til 3 timer om dagen

☐3 eller flere timer om dagen

1. … gå hurtigt uden at bære noget

☐Ingen

☐Mindre end 1/2 time om dagen

☐1/2 til1 time om dagen

☐1 til 2 timer om dagen

☐2 til 3 timer om dagen

☐3 eller flere timer om dagen

Har du nogen kommentarer til spørgskemaet kan du angive dem her:

_____________________________________________________________________________________________________________________________________________________________________________________________________________________________________________________________________________________________

# Discussion

In this study, a three-step dual translation approach was used to translate and culturally adapt the English version of the self-administered PPAQ into Danish. The target group assessed the questionnaire as comprehensible, relevant to their situation and easy to fill out. The questionnaire could be completed in 10 minutes.

## Dual-panel versus forward and back translation

The dual-panel approach was used to produce a single high-quality translation (17). Previously, the forward and backward translation method has been common for translations of self-administered questionnaires (17). However, as a translation method, the dual-panel approach is found to be superior to forward and backward translation when it comes to avoiding misinterpretations despite impressions of high-quality translations (17,18). Using the dual-panel approach may be more comprehensive because of the three steps during which both bilingual laypeople and the target group are presented with the questionnaire. Also, as the target group is included in the dual-panel process, the translation can become more targeted towards the end-user and their preferences. On the other hand, this can result in a questionnaire that differs disproportionately to the original questionnaires, therefore, challenging a cross-border comparison.

Even though panel 1 and panel 2 included different participants with different educational backgrounds, both panels preferred that the the three items i.e.; 1)"Sitting and using a computer or writing while not at work", 2)"Watching TV or a video" and 3)"Sitting and reading, talking or on the phone while not at work" (11) were combined into one item. The two panels found it challenging to differentiate between time spent on one device compared to another. Consequently, following step three and based on target group statements, the three items were combined into one item. This opportunity to verify the questionnaire's adjustments emphasises the importance of involving a target group during translations and cultural adaptations of questionnaires. The three items were all within the same MET ranges, and, therefore, differentiation between the items has no practical implication to the responder's PA level (15).

Except for the removed item "Taking care of an older adult", the target group found PPAQ-DK2 relevant for their present situation (during pregnancy). This supports the questionnaires' face validity and the time to fill out the questionnaire was approximately 10 minutes, which is in line with other translations of the PPAQ (12,19).

## Cultural adaptations of items

All responders in the target group reported that they spent no time taking care of elderly adults. In Denmark, very few people are primary caregivers to elderly or disabled family members. The Danish welfare system is organised through public and private services and homes for elderly or disabled persons who depend on help from others for their daily living activities (20). Therefore, the item regarding "Taking care of an older adult" (11) is not relevant to the majority of pregnant Danish women. A question that has no different answers (as in this item) indicates a low level of discrimination and therefore, has little sensitivity, reducing the responsiveness of the questionnaire (21). Consequently, it was decided to remove this item in the final Danish version of the questionnaire (Appendix 2).

In Denmark, it is common to commute by bicycle, i.e. 15% of all transportation is by bike. Furthermore, young women show the highest rate for commuting on bikes (22). Therefore, pregnant women are likely to continue using cycling as a means of transport throughout their pregnancy. In the Japanese translation and cultural adaptation of the PPAQ, one additional item for "riding a bicycle for reasons other than for recreation or exercise (to catch a bus, go to work, or visit a place, etc.)" was added to the Japanese version of the PPAQ. Likewise, this addition of an item was justified by cultural adaptation to the Japanese transportation pattern (12). The Japanese translation's MET values were assessed as 8.0, corresponding to leisure and moderate comfort (12). During the Danish translation of the PPAQ, however, two additional items regarding commuting on bikes were added to specify the level of physical activity during cycling, i.e. light intensity corresponding to 6.0 METs and moderate-intensity corresponding to 10.0 METs (15). This considers, for example, the growing use of electric bikes in the Danish community (23), which is less energy-consuming compared to traditional pedal-powered manual cycling.

## Limitations

The adjustments of the PPAQ during the translation and adaptation to suit the Danish culture resulted in both the removal and merging of items and the inclusion of two additional items. This changes the dynamic between the different domains in the questionnaire. Similar adaptations to the original version of the PPAQ have been made during translations to other languages and cultures (12,24,25), thereby ensuring the face validity and relevance of questionnaires concerning the target groups. These cultural adaptations and variations may influence the comparability of the questionnaire internationally at the cost of strengthening the assessment of the pregnant women's physical activity levels. However, the original PPAQ was developed in an American setting decades ago, and therefore, some adaptions must be expected because of both community changes and cultural differences.

# Conclusion

This study translated and cross-culturally adapted the original American-English version of the PPAQ to suit the Danish culture and language. The PPAQ-DK2 was found to be relevant, easy to comprehend, and quick to complete. Researchers may use the questionnaire to assess physical activity levels among pregnant women. The questionnaire may also be a framework for conversations between pregnant women and their healthcare-professionals in clinical practices concerning physical activity and how to conform to national recommendations on physical activity during pregnancy (10). Further investigations are recommended to validate and investigate the psychometric measurement properties of the Danish version of the questionnaire.

# References

1. Sundhedsstyrelsen. Fysisk aktivitet og evidens: Livstilssygdomme, folkesygdomme og risikofaktorer mv.: Et opslagsværk til rådgivning og pressedækning. København: Sundhedsstyrelsen; 2006. 26 s., med bibl.

2. Dempsey JC, Sorensen TK, Williams MA, Lee I, Raymond S, Dashow EE, et al. Prospective Study of Gestational Diabetes Mellitus Risk in Relation to Maternal Recreational Physical Activity before and during Pregnancy. 2004;159(7):663–70.

3. Sorensen TK, Williams MA, Lee I, Dashow EE, Thompson M Lou, Luthy DA, et al. Recreational Physical Activity During Pregnancy and Risk of Preeclampsia. 2003;1273–80.

4. Evenson KR, Siega-Riz AM, Savitz DA, Leiferman JA, Thorp JMJ. Vigorous leisure activity and pregnancy outcome. Epidemiology. 2002 Nov;13(6):653–9.

5. Hatch MC, Shu XO, McLean DE, Levin B, Begg M, Reuss L, et al. Maternal exercise during pregnancy, physical fitness, and fetal growth. Am J Epidemiol. 1993 May;137(10):1105–14.

6. Solomon CG, Willett WC, Carey VJ, Rich-Edwards J, Hunter DJ, Colditz GA, et al. A prospective study of pregravid determinants of gestational diabetes mellitus. JAMA. 1997 Oct;278(13):1078–83.

7. Juhl M. Physical Exercise During Pregnancy and Reproductive Outcomes. 2009; Available from: http://www.si-folkesundhed.dk/upload/rapport_phd_mette.pdf

8. Broberg L, Ersbøll AS, Backhausen MG, Damm P, Tabor A, Hegaard HK. Compliance with national recommendations for exercise during early pregnancy in a Danish cohort. BMC Pregnancy Childbirth [Internet]. 2015;15:317. Available from: http://www.pubmedcentral.nih.gov/articlerender.fcgi?artid=4661949&tool=pmcentrez&rendertype=abstract

9. Borodulin KM, Evenson KR, Wen F, Herring AH, Benson AM. Physical activity patterns during pregnancy. Med Sci Sports Exerc. 2008;40(11):1901–8.

10. Mottola MF, Davenport MH, Ruchat SM, Davies GA, Poitras V, Gray C, et al. No. 367-2019 Canadian Guideline for Physical Activity throughout Pregnancy. J Obstet Gynaecol Canada. 2018;40(11):1528–37.

11. Chasan-Taber L, Schmidt MD, Roberts DE, Hosmer D, Markenson G, Freedson PS. Development and validation of a pregnancy physical activity questionnaire. Med Sci Sports Exerc. 2004;36(10):1750–60.

12. Matsuzaki M, Haruna M, Nakayama K, Shiraishi M, Ota E, Murayama R, et al. Adapting the Pregnancy Physical Activity Questionnaire for Japanese Pregnant Women. JOGNN - J Obstet Gynecol Neonatal Nurs. 2014;43(1):107–16.

13. Sattler MC, Jaunig J, Watson ED, Van Poppel MNM, Mokkink LB, Terwee CB, et al. Physical Activity Questionnaires for Pregnancy: A Systematic Review of Measurement Properties Key Points. 2018 [cited 2018 Oct 26];48:2317–46. Available from: https://doi.org/10.1007/s40279-018-0961-x

14. World Medical Association Declaration of Helsinki: ethical principles for medical research involving human subjects. JAMA. 2013 Nov;310(20):2191–4.

15. Ainsworth BE, Haskell WL, Whitt MC, Irwin ML, Swartz AM, Strath SJ, et al. Compendium of physical activities: An update of activity codes and MET intensities. Med Sci Sports Exerc. 2000;

16. Chasan-Taber L. Pregnancy Physical Activity Questionnaire Assessment. DAPA Meas Toolkit [Internet]. 2004;0–2. Available from: https://dapa-toolkit.mrc.ac.uk/pdf/pa/PPAQ_instructions_1.pdf

17. Swaine-Verdier A, Doward LC, Hagell P, Thorsen H, McKenna SP. Adapting quality of life instruments. Value Heal [Internet]. 2004;7(SUPPL. 1):S27–30. Available from: http://dx.doi.org/10.1111/j.1524-4733.2004.7s107.x

18. Hagell P, Hedin P-J, Meads DM, Nyberg L, McKenna SP. Effects of method of translation of patient-reported health outcome questionnaires: a randomized study of the translation of the Rheumatoid Arthritis Quality of Life (RAQoL) Instrument for Sweden. Value Heal J Int Soc Pharmacoeconomics Outcomes Res. 2010;13(4):424–30.

19. Chandonnet N, Saey D, Alméras N, Marc I. French Pregnancy Physical Activity Questionnaire Compared with an Accelerometer Cut Point to Classify Physical Activity among Pregnant Obese Women. PLoS One. 2012 Jun 11;7:e38818.

20. Social policy in Denmark. Kbh.: Ministry of Social Affairs and Integration; 2011. 31 sider.

21. Fayers, Peter M. and Machin D. Questionnaire Development and Scoring [Internet]. Quality of Life. 2000. p. 135–53. (Wiley Online Books). Available from: https://doi.org/10.1002/0470846283.ch7

22. Anderson MKA. Characteristics of trips and travellers in private and public transportation in the danish travel survey data [Internet]. Trafikdage på Aalborg Universitet online. 2010. (Mobilitet og adfærd; vol. 2010). Available from: http://www.trafikdage.dk/td/papers/papers10/406_MarieKAnderson.pdf

23. Engmann TS. Elcykler vinder frem på det danske cykelmarked - Danmarks Statistik [Internet]. 2019 [cited 2020 Nov 4]. p. 1. Available from: https://www.dst.dk/da/Statistik/bagtal/2019/2019-05-10-elcykler-vinder-frem-paa-det-danske-cykelmarked#

24. Papazian T, El Osta N, Hout H, Chammas D El, El Helou N, Younes H, et al. Pregnancy physical activity questionnaire (PPAQ): Translation and cross cultural adaption of an Arabic version. PLoS One [Internet]. 2020 Mar 30;15(3):e0230420. Available from: https://doi.org/10.1371/journal.pone.0230420

25. Coll-Risco I, Camiletti-Moirón D, Acosta-Manzano P, Aparicio VA. Translation and cross-cultural adaptation of the Pregnancy Physical Activity Questionnaire (PPAQ) into Spanish. J Matern neonatal Med Off J Eur Assoc Perinat Med Fed Asia Ocean Perinat Soc Int Soc Perinat Obstet. 2019 Dec;32(23):3954–61.
